# Modern Sociology

**Published:** 1899-04-22

**Authors:** 


### Modern Sociology.

### THE QUESTION OF CORPORAL PUNISHMENT FOR CRIMINALS.

A letter has come under our notice, written by the secretary of the prison reform department of the Humanitarian League, in which he assumes that there is a more general reaction in favour of corporal punishment for criminals than we believe to be the case. He founds this opinion, to some extent, apparently, on the fact that the women suffragists of the Edinburgh National Society have memorialised the Government on the subject. At present the punishment of the lash has no place whatever in the criminal procedure of either Scotland or Ireland—its introduction into those countries would be a totally new departure. We must be allowed to say that with all due appreciation of the philanthropy of the Scottish women suffragists, we doubt their being in a position to understand all the bearings of this very wide and serious question. We may instance for example their demand that the infliction of the Cat should be awarded to men for assaults on their wives, whereas we have heard a very astute governor of a prison deliver an opinion—shared, we believe, by all qualified to judge—that the crime of wife beating is precisely the one to which, more than all others, the punishment of flogging ought not to be awarded. The reasons are obvious. The provocation which a sober working man often receives from a drunken wife is frequently such as human nature cannot bear. He comes in hungry and fatigued from a hard day's labour, as the family bread winner, to find his wife in the public-house—his neglected children crying round a fireless hearth, and the food he had left for their support sold by their mother to procure the means of intoxication for herself. The indignant passion which gives vent to its fury in blows is almost inevitable; and there is a more sinister danger connected with such cases. A vindictive, spiteful woman, knowing that by denouncing her husband for an assault she can obtain an ample revenge by his suffering under the lash, is very likely to provoke him to use violence for the very purpose of producing this result. Such cases have not been rare in the past.

To turn, however, to the general question of a retrograde movement in favour of the severity and frequency with which corporal punishment was awarded to many offences in the earlier years of this century, we do not agree with the writer of the letter that any such desire exists among those competent to judge. The present Home Secretary is known to be strongly opposed to the use of flogging under any circumstances, and so are many of the Judges. The infliction of the birch on mischievous boys is a totally different matter, and may probably be reasonably approved by most persons in a position to award such sentences; but the flogging of adults involves a serious degradation which is keenly felt, even by the most abased natures; and formerly it was executed with a degree of cruelty which would not be considered admissible in the present day.

We believe that the most generally accepted opinion among those who have the means of studying this painful subject is against the total abolition of corporal punishment for adults. It is the one award for crime which our hardened ruffians dread the most. The possibility of being sentenced to it does to some extent restrain them from acts of brutal violence, but we do not think that our humanitarian writer need be the least afraid of a return to the wholesale use of that means of correction which obtained some fifty years ago. The Edinburgh ladies who, in defence of the feebler members of their sex, demand its introduction to Scotland and more frequent application in England form part of that large-hearted, though undiscriminating, public who of late have indulged so vigorously in the cry for prison reform. As these benevolent agitators did not always possess the information and experience which qualified them to speak on the subject, the result of their efforts has not been considered altogether satisfactory by those who were entitled to go behind the scenes in prison life. But it seems to us that they have achieved two separate changes in the general conditions of prisons and criminals, of which we will leave our readers to judge for themselves, without comment. First, there has of late been an undoubted increase in the number of persons who break windows and commit other small offences for the express purpose of making their way into the prison. A kindly magistrate, dealing lately with one such misdemeanant, took the trouble to enquire of him how it came to pass that he wished to be committed to prison; he answered with perfect candour that it was because he greatly preferred it to the workhouse, which would have been the alternative for him in his condition of destitution. Being further questioned as to details, he said that the food in prison was much more satisfactory than that to be obtained in the Union and that he would, in fact, be better cared for there in every way. That such opinions are due in great measure to the softening of prison conditions, which has been the result of the cry for reform, can hardly be doubted; but it may be questioned whether our legislators intended to turn her Majesty's prisons into a species of improved workhouses. The second change which appears to have been due to this cause has been the great accumulation of brutal crimes which the newspapers have recorded of late—murders of little children, prolonged ill-usage of helpless women, and other dastardly deeds have been described constantly. There is also one especial crime—that of procuring abortion—which has been increasing to an alarming extent; as it can be perpetrated more or less secretly, most persons have no idea of the extent to which it prevails all over the country, and how little it is considered even an offence in any degree by those who generally are concerned in it. On this subject the whole country has reason to be thankful to Judge Darling for his recent wise and equitable pronouncement on the sentences awarded to such crimes. In cases where the result has been fatal it comes under the head of Wilful murder, and the perpetrator has therefore inevitably been condemned to death; but this sentence, as the Judge pointed out, was a species of mockery, as it was never for a moment intended that it should be carried out. He insisted most rightly that while a heavy penalty of some kind should attach to this manner of destroying life, it should be one which could in all cases be duly enforced.

It is to be hoped that there will now be a lull in the active interest taken by unofficial persons in prison affairs; those really in authority may be trusted to act both with humanity and justice.

